# Disjoint components of manifest time: Commentary: Physical time within human time

**DOI:** 10.3389/fpsyg.2023.1097454

**Published:** 2023-05-25

**Authors:** Valtteri Arstila

**Affiliations:** ^1^Department of Philosophy, University of Helsinki, Helsinki, Finland; ^2^Department of Philosophy, University of Turku, Turku, Finland

**Keywords:** time, manifest time, time perception, temporal experience, time consciousness

## Introduction

Our everyday life—encounters with “moderate-sized specimens of dry goods” (Austin, 1962, p. 8)—gives rise to manifest time, the total of temporal phenomena as they appear to us and are rationalized by us. It depicts time as flowing, the present moment as unique, and that world includes various dynamic events. Physics, and especially spacetime cosmologies, on the other hand, suggest that time is static, does not flow, and that the present moment is not unique. How to reconcile these two conceptions of time gives rise to the “two times problem”.

One response is to maintain that there is no real problem because the two conceptions concern distinct domains. This response is echoed by Buonomano and Rovelli (2023). They argue that physical time and time in neurosciences emerge from different scientific domains, which concern different and quite clearly partitioned research lands. Another option, though still compatible with the first response, is to take (Smart, 1980, p. 10) challenge seriously and try to “give some sort of explanation of how (the illusion of the flow of time) arises.”

Gruber et al. (2022) adopt the latter approach and ground their explanation on Hartle's Information Gathering and Utilization System (IGUS). An IGUS captures images of its surroundings and is “conscious” of only the last image. The previous images figure in conscious states as part of the schema of the environment in which the latest image is situated. These differences explain how notions of past, present, and future emerge from the way in which IGUS processes information. Nonetheless, this provides only a partial answer to Smart's challenge since it is not evident that it accounts for the dynamicity of the purported flow of time in manifest time, and it is silent about other phenomena related to manifest time.

The authors address this shortcoming by making two general claims. First, a human model IGUS is augmented with add-ons (“gadgets”) that deal with the remaining phenomena. The proposal includes many gadgets, presumably one for each separate phenomenon. Second, they put forward the *dualistic model* of manifest time. The duality here refers to the claim that all components of manifest time have illusory and veridical aspects. For example, there are veridical experiences of (neural) temporal order and corresponding illusory experiences of causational temporal order. The former refers to the order in which experiences succeed each other, and the latter refers to illusions that our actions succeed our decisions (i.e., that we have free will and “are in charge”). It is worth noting that the expressions “veridical experience” and “illusory experience” are somewhat misleading. On the one hand, the outputs of gadgets include both perceptual and cognitive components. Thus, “experiences” refer to phenomenal states but also what the authors call “cognitions”, states such as duration judgments. On the other hand, veridical experiences concur with accepted physics; illusory experiences do not. According to Gruber et al.'s (2022) definition, thus, illusory experiences are not illusions merely because they provide false information but because they cannot provide the correct information in the first place. They cannot do so, for the false information they provide contradicts the laws of physics. This is an uncommon definition because, in usual illusions, we perceive things that we can also perceive correctly in other circumstances (e.g., colors, shapes, lengths of lines).

## No internal clock nor specious present

The dualistic model is, in my view, empirically plausible but also more controversial, and needs more justification and conceptual clarification than may first appear. Consider being an official in a running competition. You start the clock at the beginning of the race and take each contestant's time. Based on the timing, you know how long each contestant ran (duration) and their placement (temporal order). There is also a sense in which one could say that time was passing, one tick at a time, during the race (flow of time). In this example, various temporal phenomena are somehow related to the operation of a single clock. The idea of humans exhibiting a similar internal clock mechanism has been popular in time perception literature (Hoagland, 1935; Treisman, 1963; Gibbon, 1977; Wearden, 1991).

An IGUS augmented with gadgets operates very differently from an internal clock that grounds all temporal experiences since there are several gadgets, each (possibly) separate from the other. Nevertheless, for a human model IGUS, this characteristic receives support from more recent empirical literature. Indeed, as already argued by Pöppel (1988), different aspects of subjective time are due to separate mechanisms. Moreover, nowadays the idea of an internal clock even as regards duration perception is contested, and there is increasing evidence that duration judgments and the passage of time judgments are dissociated (Wearden, 2015; Droit-Volet and Wearden, 2016; Jording et al., 2022). Finally, visual change and motion perception, for example, rest on distinct mechanisms (for discussion on this point, see Arstila, 2018). Overall, concurring with the proposal at hand and with Buonomano and Rovelli (2023) as well, we can say that there are good reasons to hold the disunity of subjective time (Lloyd and Arstila, 2014).

However, the augmented IGUS within the dualistic model of manifest time results in a controversial account of temporal experiences (e.g., change, motion, succession). By far the most popular way of explaining temporal experiences rests on the notion of the specious present. In these explanations, our experiences appear as having temporally extended and structured contents. We experience change, motion, and succession directly because those temporal features manifest in the contents of the specious present. For example, we experience succession because a specious present presents us with two sensory experiences (say, sounds) that have occurred at different times. Similar to the idea of an internal clock, temporal phenomenology is subordinate to the postulated specious present, a fundamental temporal structure of consciousness. If the specious present did not exist, we would not have temporal experiences (or so the claim goes).

Gruber et al.'s (2022) proposal challenges this received view in two ways. First, as mentioned above, an IGUS is “conscious” of only the last image it captures. Consequently, the contents of the conscious states of an IGUS do not exhibit any explicit apparent duration or temporal structure. Such a view of the temporal structure of an IGUS's conscious states is a version of snapshot models of time consciousness theories, not the vastly more popular specious present theories. More precisely, since the authors maintain that we experience the dynamicity of change and motion, their overall position comes close to the dynamic snapshot models (which they acknowledge to some extent). Roughly, these models hold that streams of consciousness consist of succeeding short-lived snapshots—like frames on a film—that appear to a subject as having no apparent duration or temporal structure and—unlike frames on a film—still include dynamic phenomenology related to change and motion, for instance (Le Poidevin, 2007; Arstila, 2016, 2018; Prosser, 2016).

Second, the received view maintains that temporal experiences depend on specious presents, a single temporal structure of conscious states (or process underlying such structure). Gruber et al. (2022) however, explain temporal experiences with gadgets, and as different temporal experiences result from mostly different gadgets, they also reject the claim that temporal experiences depend on any single mechanism or process. This position is even more unusual than the dynamic snapshot model, for only Arstila (2016, 2018) has explicitly argued for it; Le Poidevin (2007) and Prosser (2016) versions of the dynamic snapshot model still appeal to short-term memory (e.g., a common mechanism) in their explanation of some temporal experiences. Then again, if some temporal experiences are due to a common mechanism, then the proposal would be closer to that of Le Poidevin and Prosser than Arstila's.

## Reservations about the dualistic model

As one might expect, I find Gruber et al.'s (2022) overall proposal creditable and more promising than its alternatives. That said, I have reservations about the details of their dualistic model of manifest time. Let me end by providing just two examples where I think further explication and justification would be valuable.

First, there are reasons to doubt the reality of all purported components of manifest time. For example, in their theory, a discrete snapshot (of perception) is a case of veridical experience and the specious present is an illusory experience. While contrasting these two positions is common, snapshots and specious presents are typically contrasted as the purported fundamental temporal structure of consciousness. They are not experiences per se but concern the temporal structure of experiential states. It does not help to regard them as cognitions either, for it is not obvious whether we need a gadget for every correct or incorrect belief. Given that an IGUS is conscious of one image (arguably, a snapshot) at a time, it already operates based on snapshot perception. Accordingly, the need for a separate gadget for snapshots (and subsequently for specious presents) is currently unmotivated. Recent studies illustrate that one can raise the same point concerning the claim that we have illusory experiences of a unique moving present (e.g., Latham et al., 2019; Miller, 2019).

Second, one can doubt whether all components have veridical and illusory aspects. Consider, for instance, retrospective and prospective duration judgments, which are cases of veridical experiences for Gruber et al. (2022) and the corresponding illusory experiences of the speed of duration judgments. The first thing to note here is that, as mentioned above, there is evidence that duration judgments and the passage of time judgments need to be separated. Given that the two vary independently and rest on different mechanisms, what justifies pairing them in the way Gruber et al. (2022) do? But, if they are not veridical and illusory aspects of the same component, then the dualistic model of manifest time necessitates that they themselves must have veridical and/or illusory counterparts. Presumably, this requirement can be easily satisfied as to duration judgments: they could still amount to veridical experiences (cognitions) and their corresponding illusory counterpart would consist of experiences of duration. Not only are we said to experience durations (e.g., Dainton, 2000; Phillips, 2012), but the dynamicity of these experiences—there is a sense in which the tracked duration is felt as growing—suggest that they are illusory rather than veridical. In this proposal, we can understand why the two types of “experiences” are grouped together, as they are indeed closely related. Moreover, the illusory dynamicity of the experience of duration needs to be addressed in any case. The situation is different, however, with the speed of the passage of time, for both judgments and experiences of it are illusory—neither of them has a “basis in reality”. Thus, to save the dualistic model of manifest time, the authors need to give an account of the corresponding veridical experience, or admit that not all components have veridical and illusory aspects.

To sum up, Gruber et al. (2022) present a version of a human model IGUS that is augmented with several gadgets along the lines of the dualistic model of manifest time. The proposal concurs with well-established positions in time perception research and findings related to neural mechanisms underlying change and motion perception. However, due to the dualistic model, their explanation is almost the opposite of the dominant time consciousness theories. Hence, the proposal will need more justification in addition to the current exposition, since the position is met with skepticism, as demonstrated by the criticism of the dynamic snapshot models. These objections can be lessened by abandoning the requirement that all components of manifest time have veridical and illusory aspects. While this would be a deviation from the dualistic model, it could also help them concerning the unclearness of their detailed theory of manifest time.

## Author contributions

The author confirms being the sole contributor of this work and has approved it for publication.
